# Ligand and Solvent Selection for Enhanced Separation of Palladium Catalysts by Organic Solvent Nanofiltration

**DOI:** 10.3389/fchem.2020.00375

**Published:** 2020-05-05

**Authors:** Junjie Shen, Kai Beale, Ida Amura, Emma A. C. Emanuelsson

**Affiliations:** ^1^Centre for Advanced Separations Engineering, University of Bath, Bath, United Kingdom; ^2^Department of Chemical Engineering, University of Bath, Bath, United Kingdom

**Keywords:** palladium, ligand, homogeneous catalysis, organic solvent nanofiltration, separation

## Abstract

Organic solvent nanofiltration (OSN) has been widely applied to separate and recycle homogeneous catalysts, but the influence of ligand and solvent selection on the performance of OSN is not fully understood. Here we prepared four palladium (Pd) catalysts by combining palladium acetate with four ligands of different molecular weights. Morphological and functional properties of the Pd catalysts were characterized by TEM, FTIR, and NMR. OSN experiments were conducted in a lab-scale dead-end filtration rig. Two commercial OSN membranes, PuraMem S600 (PS600) and DuraMem 500 (D500), were used to separate the Pd catalysts from different organic solvents (toluene, isopropanol, butanol/water, and methanol) that are specified to be compatible with, respectively. For both membranes, the pure solvent permeance was positively related to the degree of membrane swelling induced by the solvent. The solvent permeance decreased significantly after the addition of a solute, as a result of membrane fouling and concentration polarization. For the PS600 membrane, the Pd rejection in any solvent was closely correlated to the molecular weight of the ligand, which agrees with the pore-flow model. For the D500 membrane, on the other hand, there was no conclusive link between the Pd rejection and the type of ligand. The one-way analysis of variance (ANOVA) confirmed that the separation processes in PS600 and D500 membranes were controlled by different transport models. The findings shed light on the selection of ligand and solvent in OSN in order to enhance the separation of homogeneous catalysts.

## Introduction

Homogeneous catalysis by transition metal complexes offers many advantages over heterogeneous catalysis, such as high catalytic activity, high selectivity, and negligible mass transfer limitations (De Smet et al., 2001; Van Leeuwen, 2004). Such superior performance is due to the ability of transition metals to complex with a wide range of ligands such as dendrimers, polymers and polyhedral oligomeric silsesquioxanes (POSS) (Erkey, 2011). However, the application of homogenous catalysis in the chemical industry is still scarcer compared to its heterogeneous counterpart. One major reason is the difficulty in separating and recycling the catalyst from the reaction products. Conventional downstream separation processes such as distillation, precipitation, and extraction, require intensive energy, deactivate the expensive catalyst and generate metal-rich waste streams, which are unfavorable from both economic and environmental perspectives (Cole-Hamilton, 2003; Vural Gürsel et al., 2015).

Organic solvent nanofiltration (OSN) has been developed as an attractive approach for the separation and recycling of homogeneous catalysts over the past decades (Janssen et al., 2011). OSN uses solvent-resistant membranes to separate molecules based on size exclusion, charge interaction, and solute-membrane affinity (Shen et al., 2018). Since the catalyst particles are usually relatively larger than the products, OSN allows the catalyst to be retained in the retentate. OSN can be operated without any additives, phase transition or thermal input, allowing for a direct recycle of the active catalyst from the reaction mixture (Dreimann et al., 2016). OSN can combine continuous catalytic process with catalyst recovery, achieving significant economic efficiency and process intensification (Marchetti et al., 2014). A typical example of OSN applications in homogeneous catalysis is the recovery and reuse of the high-value palladium (Pd) catalyst in carbon-carbon cross-coupling reactions (e.g., Heck, Sonogashira, and Suzuki reactions) (Nair et al., 2002; Datta et al., 2003; Pink et al., 2008; Tsoukala et al., 2012; Peeva et al., 2013; Ormerod et al., 2016).

However, in some cases, the catalyst itself is about the same size as the product, making it difficult to be separated by OSN. The ligands, which are used to stabilize the catalyst, have the added benefit of changing the size and shape of the catalyst (Janssen et al., 2011; Vural Gürsel et al., 2015). Increased retention of a catalyst by adding ligands has been reported for numerous homogeneous reactions (Brinkmann et al., 1999; Dijkstra et al., 2002; Fang et al., 2011; Kajetanowicz et al., 2013) and the solvent-solute-membrane interactions are found to play a fundamental role in determining the flux and catalyst rejection characteristics (Ormerod et al., 2013, 2016). Nevertheless, the influence of ligand properties (such as structure and molecular weight) on the transport mechanisms of homogeneous catalyst in OSN is still not fully understood.

This study aims to fill this knowledge gap by attaching a homogeneous Pd catalyst to four ligands with different molecular weights and geometries and investigating their separation in four solvents by two OSN membranes. Performance indicators including permeance and solute rejection were measured to determine the effect of ligands and solvents on catalyst rejection and to understand the mechanisms underlying these effects. This work will serve as a benchmark for evaluating OSN performance in dealing with complex homogeneous catalysis systems. The data will help identify the suitable combination of ligand and solvent for use in order to achieve effective catalyst separation and recycle. It will also shed some light on the transfer mechanisms of homogeneous catalyst through OSN membranes on a molecular level.

## Experimental

### Materials

Palladium(II) acetate (Pd(OAc)_2_) was used as the precursor for preparing active Pd catalysts. Four ligands were chosen for use in this study, namely 1,3-Bis(diphenylphosphino)propane (dppp); 1,2-Bis(diphenylphosphino)benzene (dppBz); Tri(o-tolyl)phosphine (P(o-tol)_3_); and 2-Dicyclohexylphosphino-2′,4′,6′-triisopropylbiphenyl (XPhos). Their structures and molecular weights are listed in Table 1. Each of the ligands has well-documented applications for Pd-catalyzed coupling reactions (Sigma, 2013; Li et al., 2014). All chemicals were purchased from Sigma-Aldrich, UK, and were used without further purification. Four solvents were chosen for use in this study, namely toluene, butanol, isopropanol, and methanol. The physicochemical properties of the solvents are summarized in Table 2. HPLC grade solvents were obtained from Fisher Scientific, UK. Deionized water was produced by an ELGA deionizer from PURELAB Option, USA. Butanol/water mixture (ratio 5:1) was used as a representative of aqueous solvent mixtures. Two commercially available OSN membranes, PuraMem S600 (PS600) and DuraMem 500 (D500) were purchased from Evonik, UK. Specifications of the membranes are summarized in Table 3. The PS600 membrane has a molecular weight cut-off (MWCO) of 600 Da and is compatible with non-polar solvents. The D500 membrane has an MWCO of 500 Da and is compatible with polar solvents and aqueous solvent mixtures. Considering the membrane solvent compatibility (Tables 2, 3), PS600 was used for OSN experiments in toluene, isopropanol, and methanol whereas D500 was used for OSN experiments in isopropanol, butanol/water, and methanol.

**Table 1:** Formulas, molecular weights, and structures of the catalyst and ligands used in this study.

**Name**	**Formula**	**Molecular weight (g mol^**−1**^)**	**Structure**
Palladium(II) acetate	C_4_H_6_O_4_Pd	224.51	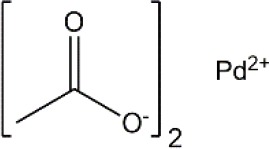
1,3-Bis(diphenylphosphino)propane (dppp)	C_27_H_26_P_2_	412.44	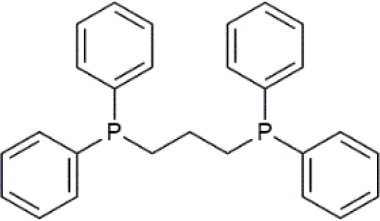
1,2-Bis(diphenylphosphino)benzene (dppBz)	C_30_H_24_P_2_	446.46	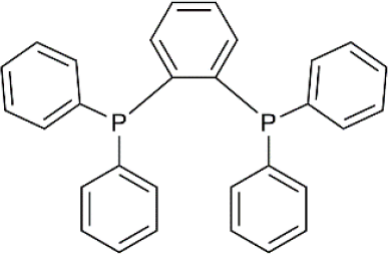
Tri(o-tolyl)phosphine (P(o-tol)_3_)	C_21_H_21_P	304.37	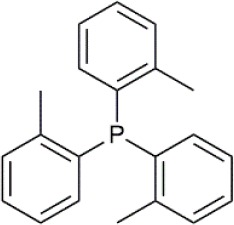
2-Dicyclohexylphosphino-2′,4′,6′-triisopropylbiphenyl (XPhos)	C_33_H_49_P	476.72	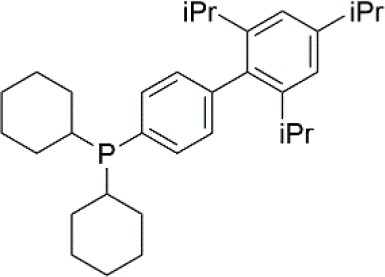

**Table 2:** Physicochemical properties of solvent used in this study (Hansen, 2007).

**Solvent**	**Molecular weight (g mol^**−1**^)**	**Density at 25 °C (kg m^**−3**^)**	**Hansen solubility parameter (MPa^**0.5**^)**	**Polarity**
Toluene	92	865	18.2	Non-polar
Butanol	74	810	23.2	Polar protic
Isopropanol	60	785	23.6	Polar protic
Methanol	32	791	29.6	Polar protic
Water	18	998	47.8	Polar protic

*The overall solubility parameter of the butanol/water mixture (ratio 5:1) was calculated using the lever rule as follows*:

*$H=\frac{5H_{B}}{6}+\frac{H_{W}}{6}=27.3\;MPa^{0.5}\;$*.

**Table 3:** Specifications of the selected commercial OSN membranes (Evonik, 2017).

**Membrane**	**PuraMem S600**	**DuraMem 500**
Material	P84 polyimide	P84 polyimide
MWCO (Da)	600	500
Maximum temperature (°C)	50	50
Maximum pressure (bar)	60	20
Useable in	Toluene, heptane, hexane, methylethylketone, methyl-isobutylketone, ethyl acetate, and more	Acetone, ethanol, methanol, tetrahydrofuran, isopropanol, acetonitrile, methylethylketone, ethyl acetate, and more
Not recommended with	Most polar and polar aprotic solvents, chlorinated solvents, strong amines	Chlorinated solvents, strong amines

### Characterization

Physicochemical properties of the catalysts were investigated by Fourier transform infrared (FTIR) spectroscopy. 0.08 mmol of Pd(OAc)_2_ and 0.16 mmol of a given ligand were dissolved in 40 mL of isopropanol. The mixtures were stirred in a Carousel 12 Plus Reaction Station (Radleys, UK) under a nitrogen atmosphere at room temperature until they were visibly homogeneous. Then the solvents were removed from the mixtures by using a rotary evaporator (Buchi Rotavapor R-215, Switzerland). The resulting powders were stored in sealed glass vials. FTIR spectra of the powders were obtained by a Spectrum 100 spectrometer with a universal ATR sampling accessory (PerkinElmer, USA). Each FTIR spectrum had 32 scans with 4 cm^−1^ resolution in the region of 650–2,000 cm^−1^.

Morphologies of the catalysts were characterized using a JEM-2100Plus transmission electron microscope (TEM) (JEOL, USA). The TEM samples were prepared by dispersing a small amount of the catalyst in ethanol, sonicating for 15 min, and placing one drop of the suspension onto 400 mesh copper grids.

The formation of the Pd-ligand complexes was further determined by nuclear magnetic resonance (NMR) spectroscopy. Standard solutions of the free ligands were prepared by dissolving 12 mg of each ligand in 0.5 mL of deuterated chloroform. Standard solutions of each Pd-ligand complex (0.08 mmol of Pd(OAc)_2_ and 0.16 mmol of a given ligand) were prepared by mixing them in 5 mL of butanol. Phosphorus-31 (31P) NMR spectra were recorded by an Avance III 500 MHz NMR spectrometer (Bruker, UK) using the solvent suppression technique.

The interactions between different solvent-membrane pairings were determined by swelling experiments. The membranes were cut into small pieces (~2 × 2 cm), weighed for an initial dry mass, and soaked in different pure solvents. The membranes samples were periodically removed from the solvent to measure the wet mass until no further increase in membrane mass was observed. The mass swelling degree (S) was calculated using Equation 1, where the initial dry mass of the membrane is *m*_*dry*_ and the swollen membrane mass is *m*_*wet*_. The swelling test was repeated three times for each membrane and the average swelling degree and the standard deviation were calculated.

(1) $$\begin{array}{l} S=\frac{m_{wet}-m_{dry}}{m_{dry}} \end{array}$$

### OSN Experiments

OSN experiments were conducted in the dead-end mode using a stainless-steel pressure cell (Sterlitech Corporation HP4750, USA). The cell has a membrane active area of 14.6 cm^2^, a maximum processing volume of 300 mL, and a maximum pressure of 69 bar. The precondition procedure was implemented by allowing 200 mL of a pure solvent to permeate through the membrane until a steady solvent flux was achieved. The membrane was preconditioned in a water bath at 25°C, pressurized with nitrogen at 40 bar, and stirred by a magnetic stirrer at 300 rpm. The permeate was collected in a measuring cylinder and was continuously weighed by an electronic balance (EK-300i, A&D Weighing, USA) under the measuring cylinder. The permeance (*P*) was determined using Equation 2, where *Q* is the permeate flow, *p* is the applied pressure and *A* is the membrane active area.

(2) $$\begin{array}{l} P=\frac{Q}{A\times p} \end{array}$$

Once preconditioned in a solvent, the membrane was used to filter 40 mL of the same solvent containing a given catalyst. The experimental conditions were the same as the preconditioning procedure. The Pd concentrations in the feed and the permeate were measured by atomic absorption spectrometry (AAS) (PerkinElmer AAnalyst 100, USA). Samples were diluted with 4-methylpentan-2-one until the Pd concentration in the samples fell within the calibration curve as drawn from the standard solutions. Each AAS measurement was carried out in triplicate and the average and the standard deviation were calculated afterwards. Experimental data were statistically analyzed by one-way analysis of variance (ANOVA) using the OriginPro 2020 software (OriginLab, USA). The significance level was set at 0.05.

## Results and Discussion

### FTIR

**Figure 1:**
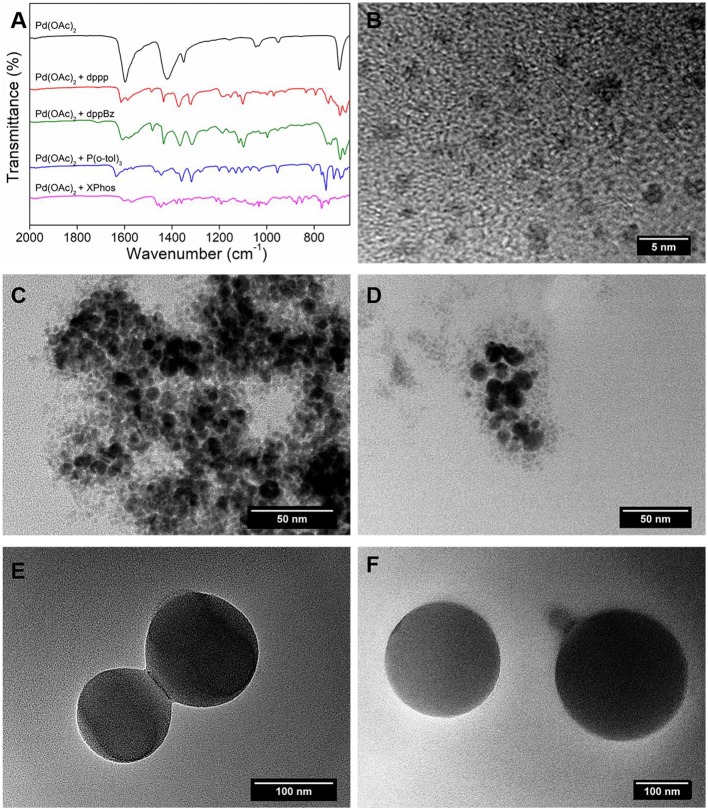
**(A)** FTIR spectra of the Pd precursor, the ligands, and the Pd-ligand complexes, **(B)** TEM image of Pd(OAc)_2_, **(C)** TEM image of Pd(OAc)_2_ + dppp, **(D)** TEM image of Pd(OAc)_2_ + dppBz, **(E)** TEM image of Pd(OAc)_2_ + P(o-tol)_3_, and **(F)** TEM image of Pd(OAc)_2_ + XPhos.

The FTIR spectra of the Pd precursor, the ligands, and the Pd-ligand complexes are illustrated in Figure 1A. The pure Pd(OAc)_2_ has characteristic peaks at 1,600 and 1,400 cm^−1^ corresponding to the C=O and C−O stretching (Pretsch et al., 2009). The Pd-ligand complexes exhibit characteristic peaks at the comparable wavenumbers to the pure substances (Daasch and Smith, 1951; Jayamurugan et al., 2009). As for the Pd(OAc)_2_ + dppp complex, the phosphorus-aryl bond at 1,590 cm^−1^ becomes more discrete and exhibits a larger peak when mixed with Pd(OAc)_2_. This can be explained as both overlapping with the more intense Pd stretching, and as a change in the local densities of the phosphorus bond, hence it can be determined that the Pd and ligand are interacting on a molecular scale (Pretsch et al., 2009). As for the Pd(OAc)_2_ + dppBz complex, the peak of the ligand's phosphorus-aryl bonds became more distributed, suggesting that interactions between the Pd and the ligand have resulted in increased stretching of these aryl bonds. As for the Pd(OAc)_2_ + P(o-tol)_3_ complex, transmittance peaks occur at 1,360 and 1,318 cm^−1^ that are attributed to a phosphorus-oxygen double bond stretch, which confirms the ability of P(o-tol)_3_ to bond to the Pd(OAc)_2_ *in-situ* (Pretsch et al., 2009). The spectra of Pd(OAc)_2_ + XPhos complex again allow for the determination of Pd-ligand bonding. The wider distribution of the phosphorus-aryl bond stretches when mixed in solution with Pd(OAc)_2_, showing significant changes in the electron distribution and thus confirming interactions between XPhos and Pd(OAc)_2_. Hence, there appears to be sufficient data to evidence that each ligand utilized in this study bonds to the Pd precursor *in-situ*.

### TEM

Figures 1B–F are the TEM images of the Pd precursor and the Pd-ligand complexes, illustrating how the ligand molecules affect the size and shape of Pd catalyst. Usually, the ligand binds to the metal catalyst and activates it by changing its oxidation state. The TEM results show that the ligand also affects the crystallinity and the tendency of Pd catalyst to form clusters and aggregates. The TEM images of the ligands alone can be found in Figure S1. The pure Pd(OAc)_2_ has the smallest particle with an average diameter of 2 nm. The Pd(OAc)_2_ particles tend to be evenly distributed in a solvent. However, when ligated with dppp and dppBz, the particles appear to agglomerate and form large clusters, significantly increasing the size of the catalyst. P(o-tol)_3_ and XPhos interact with the Pd(OAc)_2_ particles in a different way than dppp and dppBz. Instead of forming irregular clusters, they form large spherical particles with Pd(OAc)_2_, which are over 100 orders of magnitude larger than the pure Pd(OAc)_2_ particles. Hence, the different ligands result in different sizes and shapes of the Pd-ligand complex, and therefore allow a comparison between their separation performances.

### NMR

**Figure 2:**
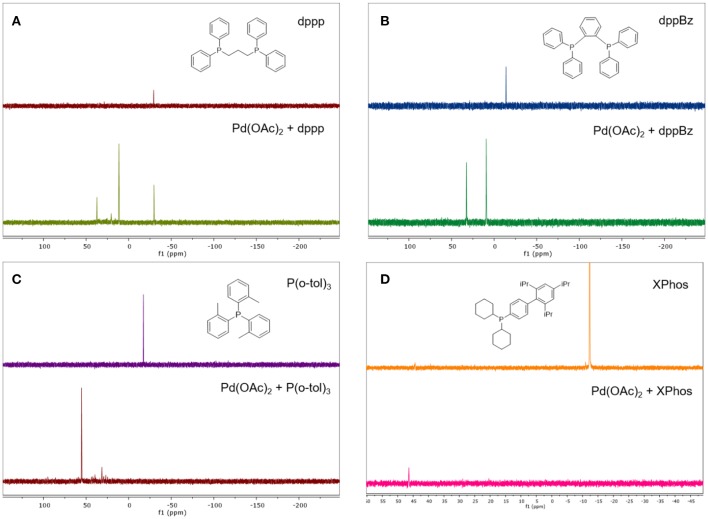
31P NMR spectra of **(A)** dppp and Pd(OAc)_2_ + dppp, **(B)** dppBz and Pd(OAc)_2_ + dppBz, **(C)** P(o-tol)_3_ and Pd(OAc)_2_ + P(o-tol)_3_, and **(D)** XPhos and Pd(OAc)_2_ + XPhos.

Figure 2 reports the 31P NMR spectra for the four free ligands and their palladium complexes. Phosphine ligands in their free form are characterized by a single peak in the low-frequency area of the spectrum between −10 and −50 ppm. For the Pd(OAc)_2_ + dppp complex, the 31P NMR spectrum presents two additional peaks in the downfield region at 30.66 ppm and 54.86 ppm. The presence of these peaks could be interpreted as an interaction of the free ligand with both the palladium and the solvent. As reported in the literature, a Pd^0^ complex is spontaneously formed from Pd(OAc)_2_ and a bidentate phosphine such as dppp. dppp is then oxidized to the hemioxide dppp(O), which can appear as a peak at around 50 ppm (Amatore et al., 2001). For the Pd(OAc)_2_ + dppBz complex, two different peaks at 9.2 and 32.4 ppm are shown in the spectrum, indicating that the two phosphorus atoms, although symmetrical in their free form, experience different magnetic fields as they interact differently with palladium. For the monodentate P(o-tol)_3_, the free ligand peak at −29.21 ppm is shifted downfield to 57.23 ppm when the Pd(OAc)_2_ + P(o-tol)_3_ complex is formed. Another small peak at 11.9 ppm appears in the spectrum which could be attributed to a conformational change of the ligand in the system. It has been reported that the P−C bonds of P(o-tol)_3_ could rotate and rearrange in two different conformations which then affect their interaction with the metal center (Widenhoefer et al., 1996). XPhos is a monodentate bulky biaryl ligand which has an intense singlet at −13 ppm. This peak is shifted to a broad singlet at 45 ppm in the spectrum of Pd(OAc)_2_ + XPhos complex. This resonance can be assigned to the XPhos-ligated Pd^II^ species Pd^II^(OAc)_2_(XPhos) (Wagschal et al., 2019). The NMR results confirm that all the four ligands can form Pd-ligand complexes with the Pd precursor *in-situ*.

### Swelling

**Figure 3:**
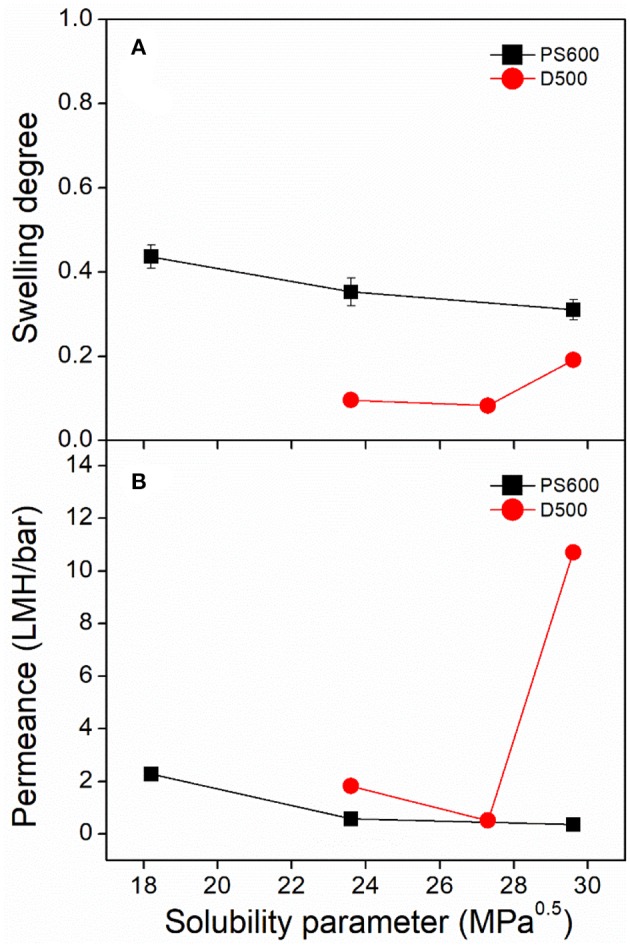
**(A)** Mass swelling degree and **(B)** pure solvent permeance of PS600 and D500 membranes as a function of the Hansen solubility parameter.

The structure and stability of a membrane can be significantly affected by swelling (Razali et al., 2017). Therefore, membrane swelling in a solvent was studied before the membrane was used for OSN. Figure 3 illustrates the mass swelling degree and the pure solvent permeance of PS600 and D500 membranes as a function of the Hansen solubility parameter listed in Table 2. For the same type of membrane, the order of pure solvent permeance mirrored the order of mass swelling degree. Specifically, the mass swelling degree of PS600 decreased from 0.43 to 0.30 as the Hansen solubility parameter increased from 18.2 to 29.6 MPa^0.5^, while its pure solvent permeance showed a similar decreasing trend from 2.28 to 0.36 LMH/bar. The mass swelling degree and the pure solvent permeance of D500 both decreased as the Hansen solubility parameter increased from 23.6 to 27.3 MPa^0.5^ and then increased sharply as the Hansen solubility parameter increased further to 29.6 MPa^0.5^. These results suggest a strong correlation between the degree of swelling and solvent permeance of OSN membranes, which is in good consistency with previous studies that found swelling caused the formation of larger channels in the polymer matrix and thus increased solvent permeance (Dijkstra et al., 2006; Marchetti et al., 2014; Shen et al., 2018).

### Permeance

**Figure 4:**
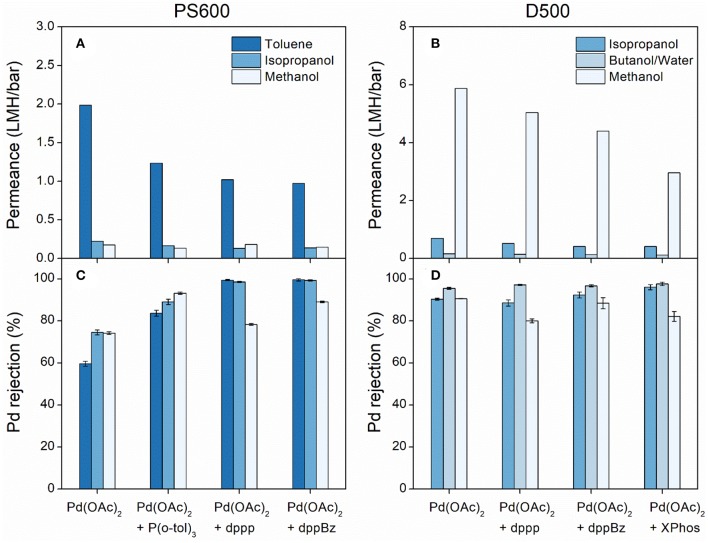
**(A)** Solvent permeance of PS600, **(B)** solvent permeance of D500, **(C)** Pd rejection of PS600, and **(D)** Pd rejection of D500 as a function of ligands.

Figures 4A,B plot the solvent permeance of PS600 and D500 membranes against different ligands. When comparing to Figure 3, both membranes showed that the pure solvent permeance was greater than the solvent permeance of Pd catalyst, indicating a negative impact of the molecular weight of the solute on the permeance. This is likely caused by fouling of the membrane by the solute particles in conjunction with concentration polarization and an increase in osmotic pressure (Davey et al., 2016). According to the ANOVA results showed in Tables S1, S2, for both membranes the effect of the ligand on solvent permeance of Pd catalyst was not statistically significant (*P* > 0.05). Regardless of the ligand type, the solvent permeance of Pd catalysts was in the same order as that of the pure solvent permeance. This suggests that, unlike the ligand, the solvent had a significant effect on the permeance. This conclusion was further supported by the ANOVA results (Tables S3, S4) where the *P*-values were far below 0.05. For the PS600 membrane, toluene had the highest permeance (0.97–1.98 LMH/bar), while isopropanol and methanol had similarly low permeance (0.13–0.22 LMH/bar). For the D500 membrane, by contrast, methanol exhibited the highest permeance (2.96–5.87 LMH/bar), isopropanol had much lower permeance (0.41–0.69 LMH/bar) and butanol/water mixture had the lowest permeance (0.11–0.16 LMH/bar). In summary, both isopropanol and methanol appeared unsuitable for use with PS600 membrane due to the low permeance, while the butanol/water mixture not suitable for D500 membrane.

### Pd Rejection

Figures 4C,D displays the Pd rejection of PS600 and D500 membranes as a function of ligands. For PS600, the addition of a ligand in all cases resulted in greater Pd rejection than just the use of pure Pd(OAc)_2_. When dissolved in isopropanol and toluene, the Pd rejection of PS600 showed a clear positive correlation against the molecular weight of the solute particles. The Pd rejection in toluene, particularly, increased from 59.6% using the pure Pd(OAc)_2_ to >99.5% rejection using a Pd(OAc)_2_ + dppBz complex. When dissolved in methanol, the results again showed that the use of ligand can be beneficial, however, there was no linear correlation between Pd rejection and molecular weight, with rejection peaking at 93.1% using a Pd(OAc)_2_ + P(o-tol)_3_ complex. This may be because the molecular weight is not the only factor that impacts Pd rejection. Other factors such as the shape of particles and the degree of agglomeration should also be considered. The *P*-values from the ANOVA results (Tables S5, S7) demonstrated that the effect of the ligand on Pd rejection was significant, while the effect of solvent was insignificant. Based on the above observations, it can be deduced that Pd separation by PS600 was governed by a pore-flow model based on size exclusion (Geens et al., 2006; Stawikowska and Livingston, 2012; Marchetti et al., 2014). The catalyst clusters observed in the TEM images could explain the high rejections observed using dppp and dppBz since the larger clusters were rejected more readily than the smaller particles in the pore-flow model.

Conversely, D500 yielded little conclusive evidence for or against the use of ligands to increase catalyst rejection. In each solvent, high rejections (>90%) were achieved with the pure Pd(OAc)_2_ solution, whereas the lowest rejections in isopropanol and methanol were observed when a ligand was used, suggesting that the use of ligand does not increase the rejection by this membrane. The *P*-values in Tables S6, S8 showed that for D500, the effect of the ligand on Pd rejection was not significant, while the effect of solvent on Pd rejection was significant. This is the exact opposite of PS600. Therefore, solute transport across D500 appears to be governed more by the solution-diffusion model which is mainly determined by solvent polarity and polymer swelling (Silva et al., 2005; Ben Soltane et al., 2013; Marchetti et al., 2014). Transport across the membrane is hence determined by the solubility of the Pd-ligand complexes in the membrane surface, and it is, therefore, likely that the molecular weight of these complexes has little bearing on their rejection profiles.

It is clear from the rejection data that the rejection of an OSN membrane is not uniquely based upon a molecular weight difference between the solute and the manufacturer's stated MWCO since each Pd-ligand complex should achieve >90% rejection in both membranes, regardless of the solvent. The results support previous studies which found that the MWCO of a membrane may not give sufficient information on its separation performance (Toh et al., 2007; Marchetti et al., 2014; Xu et al., 2017). Hence, it should be considered that the ligation of the Pd catalyst changes its chemical composition such that its transport across the membrane is altered.

## Conclusions

Firstly, this study found through TEM that the addition of ligand molecules to the Pd catalyst increases the molecular weight and produces “clusters” of molecules due to the agglomeration of organo-Pd molecules. FTIR and NMR data shows evidence for the formation of Pd-ligand bonds through changes in the spectra of the ligands in the presence of a catalyst.

Secondly, this study tested the permeance of two commercially available OSN membranes, namely PS600 and D500, in various pairings of ligand and solvent. The pure solvent permeance of both membranes was positively correlated with the swelling degree because swelling enlarged the channels in the polymer matrix. The addition of a solute, regardless of its type, decreased the solvent permeance due to membrane fouling and concentration polarization. The ANOVA results revealed that the ligand had an insignificant effect on the permeance, while the solvent had a significant effect on the permeance.

Finally, this study evaluated the Pd rejection of PS600 and D500 membranes in different ligands and solvents. The PS600 membrane exhibited a strong positive correlation between the Pd rejection and the molecular weight of the solute, with the maximum rejection of 99.5% observed with a Pd(OAc)_2_ + dppBz complex dissolved in toluene, much higher than the rejection of pure Pd(OAc)_2_. This suggests that solute transport across PS600 was relatable to a pore-flow model since particle agglomeration contributed to the high rejection. By contrast, the D500 membrane showed no conclusive link between the rejection and the molecular weight. In fact, the lowest rejections in isopropanol and methanol were observed when a ligand was used. Hence, it is believed that the transport mechanisms of the D500 membrane align with the solution-diffusion model more closely than with the pore-flow model. The ANOVA results showed that the effect of the ligand on Pd rejection was significant for the PS600 membrane, while the effect of solvent on Pd rejection was significant for the D500 membrane. These observations support our arguments that PS600 and D500 membranes are governed by different transport models.

Overall, our results confirmed the utility of OSN in the separation of homogeneous catalysts and suggested a positive correlation between rejection and solute molecular weight for membranes that follow a pore-flow model. In the future, it would be important to investigate the impact of other properties of ligand (e.g., electronic structure and conformational properties) and solvent (e.g., surface tension and viscosity), in order to draw a conclusive link between the rejection and the type of ligand and solvent. It would also be important to investigate the use of other common organometallic catalysts, such as rhodium-based molecules, to characterize the impact of ligand addition on the rejection of these catalysts by OSN.

## Data Availability Statement

The raw data of this work were deposited to FigShare for permanent storage (https://doi.org/10.6084/m9.figshare.10299047.v1). Readers can download and reuse the data for research purpose with an acknowledgment to the authors.

## Author Contributions

JS and KB performed the experiments and analyzed the data with help from IA. JS wrote the manuscript with input from all authors. JS and EE conceived the study. All authors read and approved the manuscript.

## Conflict of Interest

The authors declare that the research was conducted in the absence of any commercial or financial relationships that could be construed as a potential conflict of interest.
