# Supplementary material for: Ligand and Solvent Selection for Enhanced Separation of Palladium Catalysts by Organic Solvent Nanofiltration
Source: Front Chem. 2020 May 5;8:375. doi: 10.3389/fchem.2020.00375 (PMC7216237; doi:10.3389/fchem.2020.00375)
Supplement: Supplementary file 1 [file Data_Sheet_1.docx]

**SUPPLEMENTARY INFORMATION**

***Ligand and solvent selection for enhanced separation of palladium catalysts by organic solvent nanofiltration***

Junjie Shen^1,2^*, Kai Beale^2^, Ida Amura^1,2^, Emma AC Emanuelsson^2^*

^1^ Centre for Advanced Separations Engineering, University of Bath, Bath, United Kingdom, BA2 7AY.

^2^ Department of Chemical Engineering, University of Bath, Bath, United Kingdom, BA2 7AY.


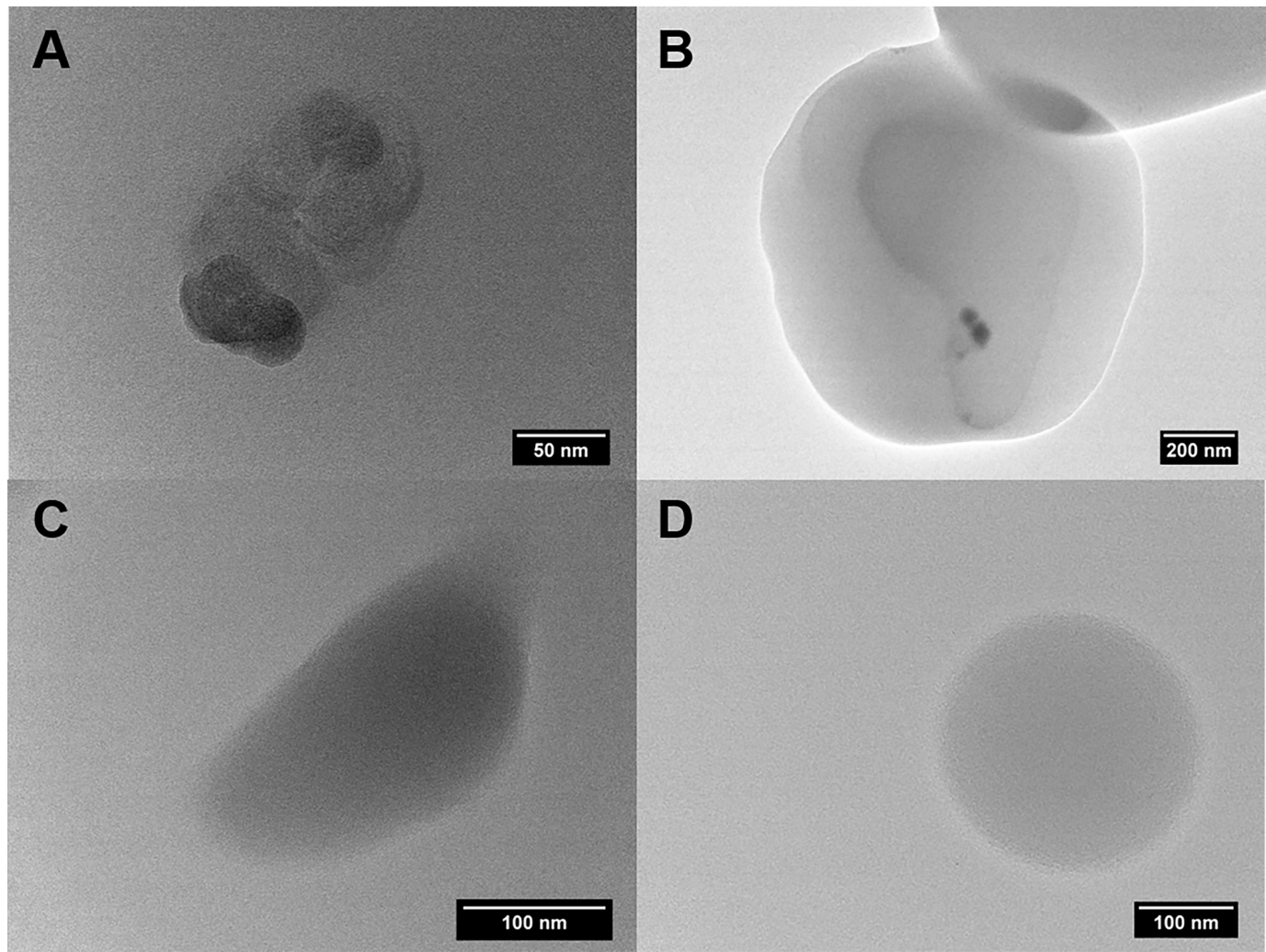


Figure S1: (A) TEM image of dppp. (B) TEM image of dppBz. (C) TEM image of P(o-tol)_3_. (D) TEM image of XPhos

Table S1: One-way ANOVA results for the effect of ligand on permeance of PS600

|  | Degree of freedom | Sum of squares | Mean squares | F value | P value |
| --- | --- | --- | --- | --- | --- |
| Between groups | 3 | 0.26871 | 0.08957 | 0.18484 | 0.90376 |
| Within groups | 8 | 3.87659 | 0.48457 |  |  |
| Total | 11 | 4.1453 |  |  |  |

Table S2: One-way ANOVA results for the effect of ligand on permeance of D500

|  | Degree of freedom | Sum of squares | Mean squares | F value | P value |
| --- | --- | --- | --- | --- | --- |
| Between groups | 3 | 1.85297 | 0.61766 | 0.09688 | 0.95958 |
| Within groups | 8 | 51.00571 | 6.37571 |  |  |
| Total | 11 | 52.85868 |  |  |  |

Table S3: One-way ANOVA results for the effect of solvent on permeance of PS600

|  | Degree of freedom | Sum of squares | Mean squares | F value | P value |
| --- | --- | --- | --- | --- | --- |
| Between groups | 2 | 3.47783 | 1.73892 | 23.4472 | 2.69733E-4 |
| Within groups | 9 | 0.66747 | 0.07416 |  |  |
| Total | 11 | 4.1453 |  |  |  |

Table S4: One-way ANOVA results for the effect of solvent on permeance of D500

|  | Degree of freedom | Sum of squares | Mean squares | F value | P value |
| --- | --- | --- | --- | --- | --- |
| Between groups | 2 | 48.27536 | 24.13768 | 47.39782 | 1.66451E-5 |
| Within groups | 9 | 4.58331 | 0.50926 |  |  |
| Total | 11 | 52.85868 |  |  |  |

Table S5: One-way ANOVA results for the effect of ligand on Pd rejection of PS600

|  | Degree of freedom | Sum of squares | Mean squares | F value | P value |
| --- | --- | --- | --- | --- | --- |
| Between groups | 3 | 1249.77097 | 416.59032 | 6.07476 | 0.01852 |
| Within groups | 8 | 548.6178 | 68.57723 |  |  |
| Total | 11 | 1798.38877 |  |  |  |

Table S6: One-way ANOVA results for the effect of ligand on Pd rejection of D500

|  | Degree of freedom | Sum of squares | Mean squares | F value | P value |
| --- | --- | --- | --- | --- | --- |
| Between groups | 3 | 29.77015 | 9.92338 | 0.23113 | 0.87222 |
| Within groups | 8 | 343.48065 | 42.93508 |  |  |
| Total | 11 | 373.2508 |  |  |  |

Table S7: One-way ANOVA results for the effect of solvent on Pd rejection of PS600

|  | Degree of freedom | Sum of squares | Mean squares | F value | P value |
| --- | --- | --- | --- | --- | --- |
| Between groups | 2 | 94.50859 | 47.2543 | 0.2496 | 0.78433 |
| Within groups | 9 | 1703.88018 | 189.32002 |  |  |
| Total | 11 | 1798.38877 |  |  |  |

Table S8: One-way ANOVA results for the effect of solvent on Pd rejection of D500

|  | Degree of freedom | Sum of squares | Mean squares | F value | P value |
| --- | --- | --- | --- | --- | --- |
| Between groups | 2 | 264.60303 | 132.30152 | 10.95939 | 0.00387 |
| Within groups | 9 | 108.64777 | 12.07197 |  |  |
| Total | 11 | 373.2508 |  |  |  |
